# Evidence of Audience Design in Amnesia: Adaptation in Gesture but Not Speech

**DOI:** 10.3390/brainsci12081082

**Published:** 2022-08-16

**Authors:** Sharice Clough, Caitlin Hilverman, Sarah Brown-Schmidt, Melissa C. Duff

**Affiliations:** 1 Department of Hearing and Speech Sciences, Vanderbilt University Medical Center, Nashville, TN 37232, USA; 2 Qntfy Corporation, Arlington, VA 22209, USA; 3 Department of Psychology and Human Development, Vanderbilt University, Nashville, TN 37235, USA

**Keywords:** audience design, common ground, perspective taking, adaptation, hippocampus, gesture, memory, language, multimodal, social cognition

## Abstract

Speakers design communication for their audience, providing more information in both speech and gesture when their listener is naïve to the topic. We test whether the hippocampal declarative memory system contributes to multimodal audience design. The hippocampus, while traditionally linked to episodic and relational memory, has also been linked to the ability to imagine the mental states of others and use language flexibly. We examined the speech and gesture use of four patients with hippocampal amnesia when describing how to complete everyday tasks (e.g., how to tie a shoe) to an imagined child listener and an adult listener. Although patients with amnesia did not increase their total number of words and instructional steps for the child listener, they did produce representational gestures at significantly higher rates for the imagined child compared to the adult listener. They also gestured at similar frequencies to neurotypical peers, suggesting that hand gesture can be a meaningful communicative resource, even in the case of severe declarative memory impairment. We discuss the contributions of multiple memory systems to multimodal audience design and the potential of gesture to act as a window into the social cognitive processes of individuals with neurologic disorders.

## 1. Introduction

When people talk, they adjust their communication for their listener. This adaptation is called *audience design* [1] and is reflected multimodally in both speech and gesture. Designing communication for a listener depends on the speaker’s awareness of the listener’s mental state and the ability to use that awareness to guide their message. The current study seeks to understand the role of the hippocampal declarative memory system in multimodal audience design. The hippocampus contributes to our ability to imagine the future and the mental states of others [2,3], as well as to the flexible use of language [4,5]. We explore whether audience design also places demands on the hippocampal memory system, where adapting communication for the listener may require reconstructing rich representations from semantic and autobiographical memory to imagine the knowledge states of others. To this aim, we examine the ability of patients with bilateral hippocampal damage and amnesia to adapt their communication in both speech and gesture for adult and child listeners. We discuss the unique affordances of speech and gesture to reflect explicit and implicit knowledge, respectively, and look for evidence of audience design in both modalities.

### 1.1. Speakers Adjust their Speech for the Listener

Speakers adjust their speech to meet the needs of their listener. They do this, in part, through the use of *common ground*. Common ground is a representation of shared knowledge and experiences that supports audience design [6]. When speakers share knowledge with their listener, their utterances are shorter and simpler [7]. This observation has been well-replicated using a collaborative referencing paradigm where speakers use increasingly shorter labels for items over multiple rounds of a barrier game as they incrementally accrue common ground with their partner [8,9,10,11]. In narrative retelling tasks, speakers use fewer words and provide less semantic information when retelling a story to a listener who has also seen the same video clip compared to a naïve listener [12]. Speakers also report fewer events and use fewer words when repeating the same story to a familiar listener compared to a new listener [13]. Multimodal approaches to studying audience design demonstrate that adaptation occurs in both speech and gesture.

### 1.2. Speakers Adjust Their Gestures for the Listener

Spontaneous co-speech gestures overlap with speech in both time and meaning and often communicate complementary information that is not present in speech. Speakers produce more meaningful gestures when their listener can see them [14], and they produce gestures in a shared common space oriented toward the listener [15]. Although speakers’ gestures reflect their own prior motoric [16] and sensory experiences [17], less is known about the extent to which our hands reflect shared knowledge and experience, above and beyond one’s own personal experience. A growing literature examines the impact of common ground on gesture use [18]. Most studies have found that speakers attenuate both their speech and gesture use in parallel as common ground increases [19,20,21,22], but see [12,23] for exceptions. Gesture reflects common ground in a variety of ways; when communicating shared knowledge, speakers produce gestures that are less complex and informative [24], smaller and less precise [20,22], and lower in the visual field [21]. 

Multimodal audience design is particularly important when adults address children, whose knowledge and mental states differ significantly from their own. Campisi and Özyürek [19] compared the narrative demonstrations adults produced when describing how to make coffee to child and adult listeners. They also manipulated common ground by including both a novice and expert adult condition. They found that participants produced more informative speech when talking to both children and novice adults compared to expert adults. However, participants increased their iconic gesture rate for the child listener only. Gestures produced for the child listener were also rated as more informative and larger than those produced in either adult listener condition. The authors concluded that iconicity is an important strategy that scaffolds communication for children and differentiates child- from adult-directed communication. 

### 1.3. Hippocampal Contributions to Language and Social Behavior

We examine multimodal audience design in patients with hippocampal amnesia. Patients with amnesia demonstrate a dissociation in memory, presenting with severe declarative memory impairments but relatively preserved nondeclarative learning [25,26,27,28]. Studying patients with amnesia provides unique insights into the role of the hippocampus in everyday flexible and adaptive social behavior [29]. Prior work has identified hippocampal contributions to language, where real-time language processing and use places demands on the hippocampal memory system [4,5]. In collaborative referential barrier games, patients with amnesia demonstrate striking learning in their acquisition of shared concise labels for referents across multiple trials; however, they do not signal shared knowledge consistently with the use of definite referents, suggesting a lack of explicit awareness of shared knowledge, and unlike neurotypical peers, their speech turns lack flexibility and acknowledgement of multiple perspectives and communal knowledge [30,31]. Patients with amnesia are also impaired at using language flexibly and creatively for verbal play in social interactions with familiar partners [32]. The hippocampus has been implicated in social cognition more broadly, including the networks dedicated to processing the thoughts and knowledge of others [33]. Together, these findings raise the possibility that the hippocampus plays a role in perspective-taking required for audience design by activating and reconstructing rich memory representations to guide language use and social behavior.

### 1.4. Current Study

To test the necessity of the hippocampal declarative memory system for multimodal audience design, we asked patients with amnesia and neurotypical peers to narrate four different procedures (e.g., how to change a lightbulb) to an adult and child listener. One possibility is that patients with amnesia will show a lack of adaptation for the child listener in both speech and gesture. Patients with amnesia gesture at lower rates during procedural and episodic discourse tasks involving the remote past [34], suggesting that memory impairments manifest multimodally in impoverished speech and gesture production. Indeed, gesture theory predicts that gesture production arises from rich mental simulations of motor, spatial, and perceptual representations [35]. Following these results, we might expect the speech and gestures produced by patients with amnesia to lack the flexibility and detail involved in designing utterances for a child listener when the task relies on reconstructing rich representations from semantic or autobiographical memory. 

An alternative possibility is that speech and gesture reflect dissociable memory systems. Gesture, unlike speech, is often produced and processed unconsciously [36] without overt attention [37,38], and can depict implicit knowledge that the speaker cannot yet verbalize [39]. If gesture is supported by neural mechanisms independent of the hippocampus (e.g., nondeclarative memory), it is possible that patients with amnesia could show adaptation in gesture without adaptation in speech. Indeed, emerging evidence suggests that gesture may be supported by nondeclarative memory and intact neural motor networks; the gestures of patients with Parkinson’s Disease, who show the oppositive pattern of memory impairments to amnesia (intact declarative memory but impaired procedural memory due to basal ganglia dysfunction), do not reflect their prior motor and perceptual experiences [40], and patients with Parkinson’s Disease produce fewer manner and first-person perspective gestures of high motion actions [41]. Similarly, despite severe declarative memory impairments, patients with amnesia benefit from gesture in their comprehension of narratives [42] and recognition memory for novel words [43]. Further, although the speech of patients with amnesia does not reflect communal knowledge, the gestures they produce do seem to be sensitive to common ground status; like neurotypical peers, patients with amnesia adapt gesture height, producing fewer visible gestures above the barrier over the course of a collaborative referencing game as common ground accrued with their partner [44]. The current study examines whether patients with amnesia show evidence of audience design in speech and/or gesture when the task requires imagining others’ mental states. 

## 2. Materials and Methods

### 2.1. Participants

Participants were 4 (one female) patients with hippocampal damage and amnesia and 11 (three female) non-brain-damaged neurotypical peers. The patients were recruited from the Patient Registry at the University of Iowa’s Division of Behavioral Neurology and Cognitive Neuroscience. All patients have non-progressive lesions. The University of Iowa Institutional Review Board approved all procedures for this study.

**Figure 1:**
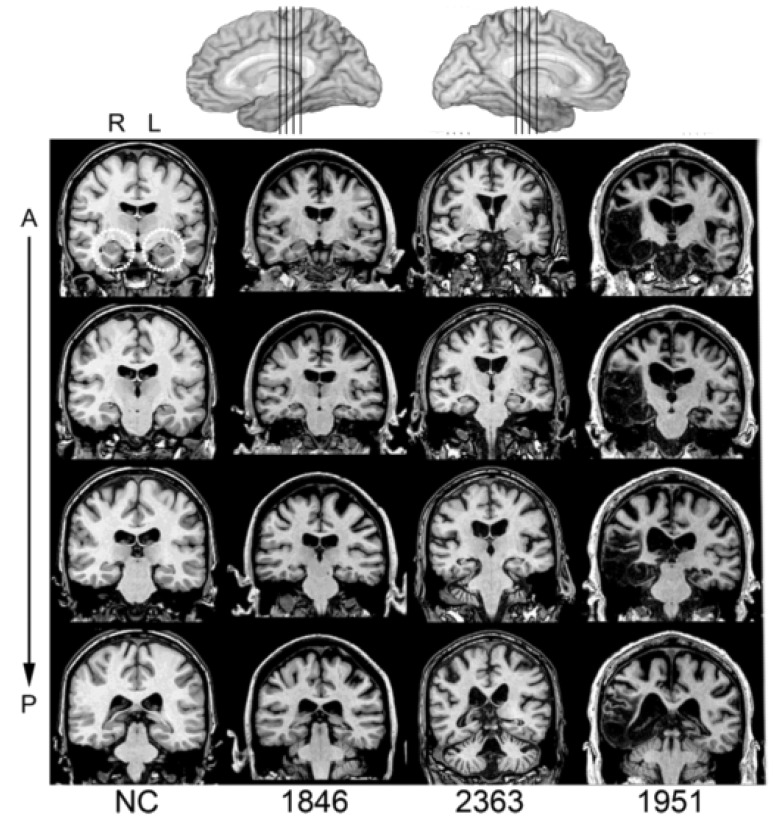
Magnetic resonance scans of hippocampal patients. Images are coronal slices through four points along the hippocampus from T1-weighed scans. Volume changes can be noted in the hippocampal region for Patients 1846 and 2363 and significant bilateral MTL damage including the hippocampus can be noted in Patient 1951. R = right, L = left, A = anterior, P = posterior, and NC = neurotypical brain.

For the amnesia group, three patients experienced anoxic/hypoxic episodes (Participants 1846, 2363, 2563), resulting in bilateral hippocampal damage, and one patient had herpes simplex encephalitis (Participant 1951), leading to more extensive bilateral medial temporal lobe damage affecting the hippocampus, amygdala, and surrounding cortices (Figure 1). High-resolution MRI analyses were conducted for the entire brain on three of the four patients [45,46]. These analyses showed hippocampal volumes significantly decreased for each patient, with the studentized residual differences in hippocampal volume relative to a matched neurotypical group reduced by 2.64, 4.23, and 8.10 z-scores for 2363, 1846, and 1951, respectively. Patient 2563 wore a pacemaker and was unable to undergo MRI examination, and thus, their damage was confirmed by computerized tomography; the damage was confined to the hippocampus. For the three anoxic patients, there was no damage to the lateral temporal lobes or anterior temporal lobes. Patient 1951 with HSE had complete loss of the right temporal pole and right temporal lobe, whereas damage to left temporal pole was confined to the medial polar cortex [47].

Tests of neuropsychological functioning revealed a severe and selective impairment in declarative memory (M = 57.6; Wechsler Memory Scale-III General Memory Index) while measures of verbal IQ, vocabulary, and semantic knowledge were within a normal range (Table 1). Patients were free of aphasia, had no motor impairments that interfered with the ability to gesture, and all showed preserved non-declarative memory ability on an extensive battery of complex perceptual-motor skill learning [25]. Neurotypical participants were 11 individuals without any neurological or psychiatric disease that were case matched to the patients with amnesia on sex, age, handedness, and education. Each patient with amnesia was matched to two or three neurotypicals participants.

### 2.2. Procedure

Participants were instructed to narrate four demonstrations of everyday activities to a listener. They did this twice: First, participants narrated demonstrations to a live adult listener—the experimenter—and then, after a half-hour delay, they narrated demonstrations again to an eight-year-old child. Due to the remote geographical locations of the patients with amnesia, it was not possible to use a live child confederate. Instead, all participants were asked to imagine they were describing the activities to an eight-year-old child. A sex-matched picture of a child was presented on a computer monitor to facilitate the interaction and ensure that the patients remembered throughout the interaction to whom they should direct their narrations. The experimenter gave the following live instructions: “I’m going to have you describe to me how to do four everyday activities. I will tell you the scenario one at a time. Describe each activity so I would be able to complete the task myself.” Then, for the second time, “I’m going to have you again describe how to do four everyday activities. This time though, I want you to imagine that you are describing them to an eight-year-old child. I will show you a picture of an eight-year-old to help facilitate this. Describe each activity so that she/he would be able to complete the task herself/himself.” Due to the small number of patients, order of the listener conditions was the same for each participant to avoid introducing additional variability into the data. 

Demonstrations included (a) how to tie a shoelace on a sneaker, (b) how to make a pot of coffee, (c) how to heat up leftovers in a microwave, and (d) how to change a lightbulb in a table lamp. All four were elicited in the same order for both listener conditions. The experimenter engaged in occasional nonverbal (e.g., nodding) or verbal backchanneling (e.g., “mmhmm”) as encouragement during both listener conditions. All demonstrations were video recorded.

#### 2.2.1. Gesture Coding

Video recordings of demonstrations were coded using ELAN video annotation software [48,49]. Gestures were identified as any hand movements that co-occurred in rhythm and/or meaning with speech. Other movements (e.g., self-adjustments) were not coded. Gestures were categorized as one of three types: iconic, beat, or deictic [50]. Iconic gestures were defined as hand movements that visually depict the shape, size, position, or movement of an object (e.g., twisting hand as if to unscrew a lightbulb). Deictic gestures were defined as hand movements that refer to the location of an object in space (e.g., pointing). Beat gestures were defined as hand movements that occur in rhythm with speech but with no semantic relation to the content (i.e., are not representative). Gestures were also coded for handedness to capture gesture informativeness. In [19], Campisi and Özyürek provide an example of a participant who uses a single-handed gesture to demonstrate, “Put the coffee in the funnel” to the expert and novice adult listeners but uses two hands to demonstrate the same step to the child listener. Thus, we looked for more two-handed gestures in the child condition as evidence of increased gesture informativeness. A gesture was considered two-handed if both hands were moving or involved in the production of the gesture (See Supplementary Materials A for Gesture Coding Guide). Gesture coding for all participants was completed by the first author. A second coder who was unaware of participant group independently coded a random selection of one demonstration per participant. Inter-rater reliability (percent agreement) for gesture identification, gesture type classification, and handedness were 91.6%, 89.8%, and 91.2%, respectively. Intra-rater reliability was calculated for the same sample of demonstrations. Intra-rater reliability for gesture identification, gesture type classification, and handedness were 95.8%, 92.7%, and 92.7%, respectively. Percent agreement for individual gestures was calculated from the total number of gestures identified by each coder, whereas reliability for gesture type and handedness was calculated from all common gestures identified by both coders at approximately the same time points. 

#### 2.2.2. Speech Coding

Each demonstration was transcribed. A total word count was calculated for each participant and demonstration to assess if participants spoke more to a specific listener. These transcripts were coded to analyze the types of details provided in the demonstrations for the two listener conditions. Ulatowska and colleagues [51] describe a framework for analyzing procedural discourse in which the basic element is the “step.” These can be subcategorized into *essential steps*, basic actions required to complete the task, and *optional steps*, those that clarify or elaborate on essential steps. Essential steps were determined a priori by a consensus of two authors. Optional steps reflect all other instructions provided. To capture additional variability in the demonstrations provided for adult and child listeners, three more categories were coded: *semantic statements*, which includes additional information about safety, world knowledge, and locations of objects; *management,* which includes organizing statements, metacognitive statements, and listener feedback; and *repetition,* which includes repeated steps and abandoned utterances (see Supplementary Materials B for Speech Coding Guide). Speech coding for all participants was completed by the first author. A second coder who was unaware of participant group independently coded 20% of the transcripts, selected at random. Inter-rater reliability across the five speech categories was 96.0% (essential steps: 98.0%; optional steps: 91.6%; semantic information: 94.9%; management: 76.5%; repetition: 76.0%). Due to the lower reliability of management and repetition categories, these were excluded from subsequent analyses.

### 2.3. Analysis

We used mixed effect regression models that predicted the dependent variable of interest as a function of group (amnesia vs. neurotypical), listener (adult vs. child), and their interaction using the lmer() and glmer() functions of the *lme4* package (version 1.1-21) [52], as well as the glmmTMB() function from the *glmmTMB* package [53] in R (R Core Team, 2021) with a linking function that was appropriate to the data structure (log for count data and logit for binary data). To interpret significant coefficients for log-linked regressions, we used incidence rate ratios (IRR). To interpret significant coefficients for logit-linked binomial regressions, we used odds ratios. The participant group was dummy coded such that the amnesia group served as the reference group. In doing so, the main effect of the listener is interpreted as the simple effect for patients with amnesia. The listener was effects coded (adult: −0.5, child: 0.5). We probed interactions between participant group and listener by reverse-dummy coding the model, setting the neurotypical group as the reference to determine the simple effect of the listener for neurotypical participants. We initially attempted to fit the models using a maximal random-effects structure [54] with random intercepts for the participant and task and random by-participant slopes to account for individual variability. When models failed to converge, we removed terms from the model, starting with random slopes for the effect of listener by participant, followed by random intercepts for task, and then participants, until they successfully converged. Results for all models are reported in Appendix A.

## 3. Results

### 3.1. Speech

#### 3.1.1. Word Count

**Figure 2:**
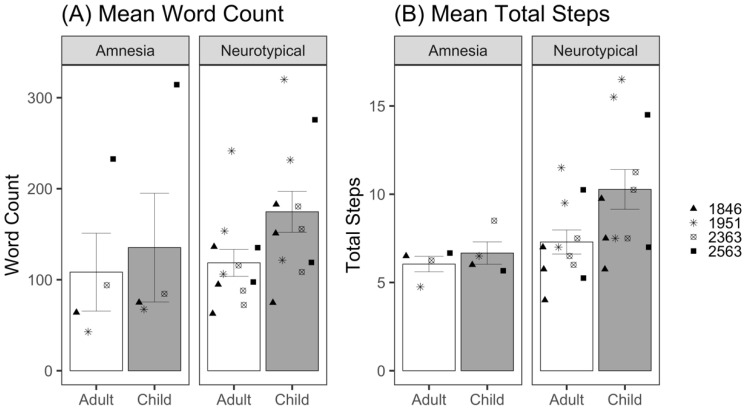
(**A**) Mean total words produced per demonstration for adult and child listeners by patients with amnesia and neurotypical participants. (**B**) Mean total steps produced per demonstration for adult and child listeners by patients with amnesia and neurotypical participants. Bars represent standard error of the mean. Points indicate mean performance of individual participants, with neurotypical participant points corresponding to the matched patient with amnesia.

Table 2 shows an example transcript of a demonstration for each listener condition by a patient with amnesia and a neurotypical participant. We modeled the number of words participants said when talking to child and adult listeners using a negative binomial distribution for count data, as our initial model using a Poisson distribution was overdispersed. There was no significant effect of listener (*β* = 0.18, *z* = 1.51, *p* = 0.13; Table A1); patients with amnesia did not significantly differ the number of words they said to the child and the adult listener (Figure 2A). The effect of group was also not significant (*β* = 0.31, *z* = 1.14, *p* = 0.26); patients with amnesia and neurotypical participants did not significantly differ in the number of words they said. The interaction between listener and group was also not significant (*β* = 0.20, *z* = 1.47, *p* = 0.14), meaning the effect of listener on word count did not significantly differ between neurotypical participants and patients with amnesia. 

#### 3.1.2. Total Steps

Total steps were calculated as the sum of essential and optional steps provided for each task by participants in each listener condition. We modeled the total number of steps provided to child and adult listeners using a Poisson distribution for count data; no overdispersion was detected. There was no significant effect of the listener (*β* = 0.12, *z* = 0.80, *p* = 0.43; Table A2); patients with amnesia did not significantly differ the number of total steps they provided to child and adult listeners (Figure 2B). There was no effect of group (*β* = 0.26, *z* = 1.55, *p* = 0.12); patients with amnesia did not significantly differ from neurotypical participants in the number of total steps they provided. The listener–group interaction additionally was not statistically significant (*β* = 0.23, *z* = 1.40, *p* = 0.16), meaning the effect of listener on total steps provided did not significantly differ between neurotypical participants and patients with amnesia.

#### 3.1.3. Speech Acts

**Figure 3:**
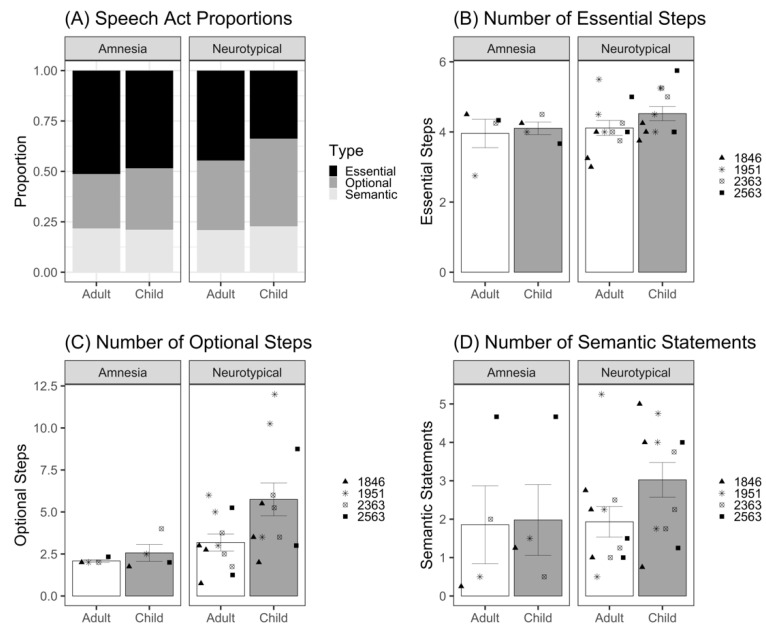
(**A**) Proportion of speech categories produced for adult and child listeners by neurotypical and amnesia groups. (**B**) Mean number of essential steps produced per demonstration for adult and child listeners by patients with amnesia and neurotypical participants. (**C**) Mean number of optional steps produced per demonstration for adult and child listeners by patients with amnesia and neurotypical participants. (**D**) Mean number of semantic statements produced per demonstration for adult and child listeners by patients with amnesia and neurotypical participants. Bars represent standard error of the mean. Points indicate mean performance of individual participants, with neurotypical participant points corresponding to the matched patient with amnesia.

We report proportions for the three coded speech act categories that met the reliability criterion (essential steps, optional steps, and semantic information) produced for adult and child listeners by both groups in Figure 3A. The neurotypical group appears to demonstrate a trade-off between essential and optional steps for adult versus child listeners. Although *essential steps* represented the largest category (45% of demonstration) for the adult listener, it accounted for only 34% of the demonstration for the child listener. In contrast, *optional steps* represented the largest category for the child listener at 43%, compared to 34% for the adult listener. These categories followed a different pattern in the amnesia group. Patients with amnesia produced mostly *essential steps* for both the adult and child listener at 51% and 49%, respectively. *Optional steps* was the next largest category for both the adult and child listener at 27% and 30%, respectively. Semantic information (safety, world knowledge) accounted for similar proportions of the demonstrations (21–23%) across both groups and listener conditions. These included descriptions of typical locations, features of objects, and safety information (e.g., “lightbulbs can be hot”). Three out of four patients with amnesia provided this kind of safety information in speech. Participant 1846 warned the child listener not to touch the hot lightbulb, participant 1951 warned both the adult and child listener not to drop the lightbulb and warned the child listener not to look directly at the light when turned on, and participant 2563 warned the child listener against leaving the leftovers in the microwave too long so as not to burn their mouth. 

To test whether there were statistical differences in these categories, we modeled the number of essential steps, optional steps, and semantic statements provided to child and adult listeners using a Poisson distribution for count data; no overdispersion was detected. For *essential steps* (Figure 3B and Table A3), there was no significant effect of listener (*β* = 0.05, *z* = 0.27, *p* = 0.79) or group (*β* = 0.07, *z* = 0.67, *p* = 0.50). The listener–group interaction was also not significant (*β* = 0.05, *z* = 0.22, *p* = 0.83). For *optional steps* (Figure 3C and Table A4), there was no significant effect of listener (*β* = 0.23, *z* = 0.97, *p* = 0.34), and the effect of group did not reach significance (*β* = 0.49, *z* = 1.66, *p* = 0.10). The listener–group interaction was also not significant (*β* = 0.36, *z* = 1.39, *p* = 0.16). For *semantic statements* (Figure 3D and Table A5), there was no significant effect of listener, (*β* = 0.17, *z* = 0.52, *p* = 0.61), group (*β* = 0.34, *z* = 0.95, *p* = 0.34), or listener–group interaction (*β* = 0.33, *z* = 0.90, *p* = 0.37) These results indicate that patients with amnesia did not significantly differ from neurotypical comparison participants in the number of essential steps, optional steps, or semantic statements that they provided. In addition, participants provided similar number of essential steps, optional steps, and semantic statements to the child and the adult listener.

As a side note, we conducted an exploratory analysis of the effect of listener on our speech measures in the neurotypical group. In the above models, we did not detect significant effects of listener on any of the speech measures in the amnesia group. The lack of significant interaction between listener and group in each of these models suggest that there is not a significant difference in the effect size betas between the amnesia and neurotypical groups. However, due to our necessarily small sample size, we caution the reader in over-interpreting this lack of significant interaction as evidence that the neurotypical participants showed no adaptation for the child listener in speech. Figure 2 and Figure 3 show that neurotypical participants produce a numerically greater adaptation in speech for the child listener compared to adult listener (e.g., more words, total steps, optional steps, semantic statements), consistent with a robust literature establishing effects of audience design in non-brain-injured individuals (described above). The aim of the current study was not to test whether neurotypical adapt their communication for their listener, but rather, to test whether patients with amnesia do. By dummy coding the amnesia group as the reference group in each model, we maximized our power to detect such effects in amnesia, at the cost of reduced power to detect differing effects in the neurotypical participants via the group–listener interaction. When we conducted post hoc statistical tests by dummy coding the neurotypical group as the reference group, we found that the effects of listener in the neurotypical group produced consistently larger beta values for speech measures than the amnesia group; Word Count: *β* = 0.37, *z* = 5.61; Number of Total Steps: *β* = 0.34, *z* = 4.70; Number of Optional Steps: *β* = 0.59, *z* = 5.66; Number of Semantic Statements: *β* = 0.51, *z* = 2.85).

### 3.2. Gesture

Neurotypical participants produced an average of 14.8 gestures per demonstration, and patients with amnesia produced an average of 10.0 gestures per demonstration (see Appendix B for variability in outcome variables by demonstration). The majority of these gestures were iconic (Neurotypical = 68%, Amnesia = 63%), followed by beat gestures (Neurotypical = 31%, Amnesia = 36%), with very few deictic gestures produced (Neurotypical = 1%, Amnesia = 1%). Due to the small number of deictic gestures, iconic and deictic gestures were collapsed into one representative gesture category. Beat gestures were considered nonrepresentative. 

#### 3.2.1. Gesture Rate

**Figure 4:**
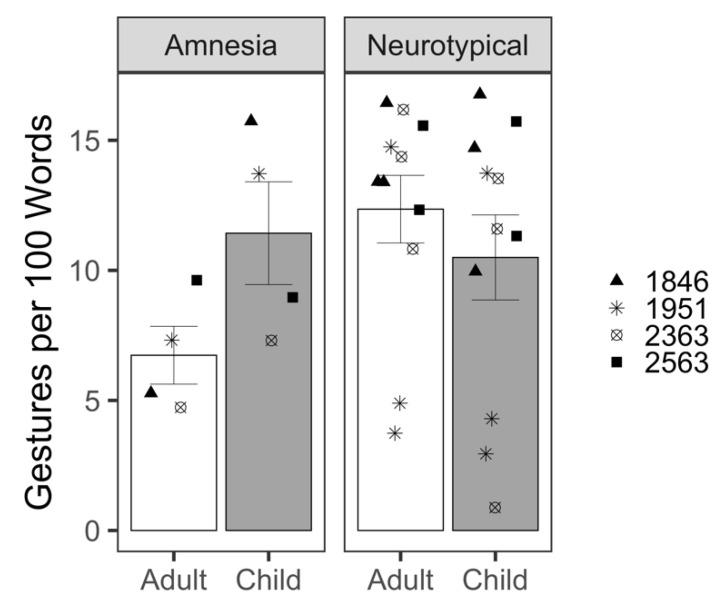
Mean gesture rate produced per demonstration for adult and child listeners by patients with amnesia and neurotypical participants. Bars represent standard error of the mean. Points indicate mean performance of individual participants. Symbols correspond to each person with amnesia and their demographically matched neurotypical peers.

Gesture rate was measured as the number of all gestures per 100 words. We modeled the rate of gesture production with a Gaussian distribution. The analysis of gesture rate revealed a significant effect of listener (*β* = 4.84, *t*(13.32) = 2.26, *p* = 0.04; Table A6); patients with amnesia produced almost five more gestures per 100 words when talking to the child compared to the adult listener (Figure 4). The effect of group was not significant (*β* = 2.34, *t*(13.14) = 0.99, *p* = 0.34), indicating that overall, patients with amnesia and neurotypical comparison participants did not significantly differ in the gesture rates they produced. The interaction between listener and group was significant (*β* = −6.70, *t*(13.16) = −2.69, *p* = 0.02). To investigate the interaction, we re-ran the model, setting the neurotypical group as the reference level; this analysis revealed no significant effect of listener on gesture rate for the neurotypical group (*β* = −1.86, *t*(12.72) = −1.46, *p* = 0.17; Table A7); unlike patients with amnesia, neurotypical participants did not produce higher gesture rates for the child compared to adult listener.

#### 3.2.2. Gesture Informativeness

**Figure 5:**
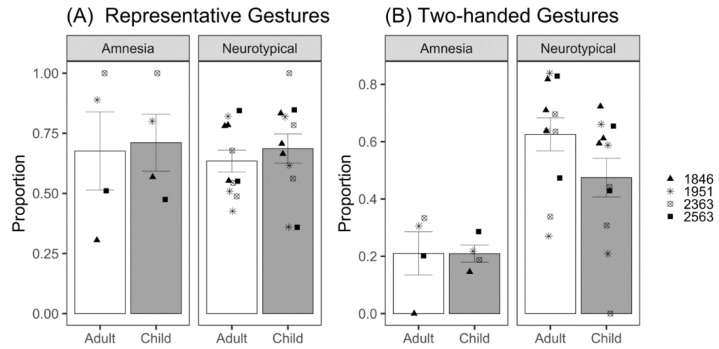
(**A**) Mean proportion of representative gestures produced per demonstration for adult and child listeners by patients with amnesia and neurotypical participants. (**B**) Mean proportion of two-handed gestures produced per demonstration for adult and child listeners by patients with amnesia and neurotypical participants. Bars represent standard error of the mean. Points indicate mean performance of individual participants. Symbols correspond to each person with amnesia and their demographically matched neurotypical peers.

To analyze gesture informativeness, we first looked at the representative gestures (iconic and deictic) participants produced to child and adult listeners (Figure 5A). We modeled the likelihood of producing a representative gesture. There was no significant effect of listener (*β* = 0.44, *z* = 1.05, *p* = 0.30; Table A8); patients with amnesia were similarly likely to produce representative gestures to both adult and child listeners. The effect of group was also not significant (*β* = −0.09, *z* = −0.16, *p* = 0.87); patients with amnesia and neurotypical participants did not significantly differ in their likelihood of producing representative gestures. The interaction between listener and group was also not significant (*β* = −0.30, *z* = −0.66, *p* = 0.51). 

Next, we looked at the probability of producing a two-handed gesture. Gesture handedness was coded as right-handed, left-handed, or two-handed. We modeled the likelihood of producing a two-handed gesture. There was no significant effect of listener (*β* = 0.42, *z* = 1.46, *p* = 0.15; Table A9); patients with amnesia were similarly likely to produce two-handed gestures to both adult and child listeners (Figure 5B). There was a significant effect of group (*β* = 1.62, *z* = 3.73, *p* < 0.001); neurotypical participants were approximately 5 times more likely to produce two-handed gestures than patients with amnesia. The interaction between listener and group was significant (*β* = −0.91, *z* = −2.90, *p* = 0.004); the lack of listener effect reveals that patients with amnesia produced similar amounts of two-handed gestures to adult and child listeners. To probe the interaction, we dummy coded the analysis to use the neurotypical group as the reference. By contrast and contrary to our prediction, neurotypical participants produced fewer two-handed gestures to the child listener (*β* = −0.49, *z* = −3.89, *p* < 0.001), with the odds of a two-handed gesture significantly 1.63 times greater for adult compared to child listeners. 

## 4. Discussion

When people talk, they adjust their communication for their listener. The present findings expand on previous work characterizing how speakers adapt their speech and gesture for the needs of a listener by exploring the neural mechanisms that support audience design. Here, we asked whether patients with hippocampal amnesia adjust their speech and gesture production when designing communication for adult and child listeners. We did not find statistical evidence that patients with amnesia (or neurotypical participants) adapted their speech for the child listener. However, patients with amnesia (but not neurotypical participants) produced gestures at higher rates for the child compared to the adult listener. Our necessarily small sample size limits our power to detect interaction effects that would allow us to determine whether neurotypical participants differed from patients with amnesia in the effect of listener on speech and gesture use. As such, we focused our discussion largely on the findings in amnesia.

### 4.1. Hippocampal Contributions to Audience Design in Speech

Patients with amnesia showed preserved ability to produce narrative demonstrations; consistent with prior work [55], patients with amnesia did not differ from neurotypical comparisons in the number of instructional steps they provided. However, patients with amnesia did not show evidence of audience design for the child listener in their speech. The number of words, total steps, essential steps, optional steps, and semantic statements they provided did not significantly differ between adult and child listener conditions. These findings suggest that the hippocampal declarative memory system may contribute to the ability to adapt speech behavior to meet listeners’ needs, and when damaged, may disrupt verbal audience design. This is consistent with prior work showing that the hippocampus plays a role in reconstructing prior knowledge and experiences in novel ways that contributes not only to remembering the past (autobiographical memory), but also to imagining the future (prospection) and the mental states of others (theory of mind) [2,3,56]. We propose that patients with amnesia in this study were unable to imagine the knowledge state of a child when demonstrating familiar tasks and use such knowledge to flexibly adapt their speech accordingly. 

That said, there are occasions when patients with amnesia do show speech adaptation; previous work investigating the role of the hippocampal declarative memory system in common ground showed that patients with amnesia do shorten their utterances for their listener when developing incremental common ground in collaborative referential barrier games [57] and even demonstrate partner-specific knowledge, differentiating new and familiar partners by using shorter labels with familiar partners and increasing the length of their descriptions for new partners [58]. Furthermore, patients with amnesia, like neurotypical participants, synchronize their speech (i.e., produce utterances that increasingly match the length of their communication partner’s utterances) over the course of dynamic conversation [59]. It is a critical question, then, why patients with amnesia would show speech adaptation in some cases but not here. In the current study, audience design did not rely on incremental common ground experience, but rather on reconstructing prior knowledge to imagine listener needs and tailor communication for the unique needs of listeners with different levels of experience. This type of audience design likely requires drawing on more generic knowledge of shared information within communities of people [60]. For example, taking the perspective of a child may require activating general schemas for what children know or reconstructing experiences from one’s own childhood or interactions with children to adjust behaviors accordingly. We suggest that it is the increased memory demands involved in imagining other’s knowledge states that drive the lack of speech adaptation in amnesia in the current study, whereas the acquisition of shared labels and conversational alignment are acquired through incremental experience with a conversation partner. The Interactive Alignment theory [61] proposes that this process is achieved through priming and is largely automatic, often based on an implicit common ground rather than direct, explicit inferring and tracking of the listener’s and speaker’s own knowledge states. Thus, some aspects of speech adaptation may be supported by non-declarative memory, while others may recruit declarative memory [30,62]. This proposal warrants further investigation.

Notably, we did not detect statistical evidence of audience design in speech for the child listener in our neurotypical group either. The effects of audience design in neurotypical speakers are well-established in the literature; people shorten their utterances when they share common ground with the listener [9], adjust the amount of detail provided to novice vs. expert listeners [7,19], and tailor speech to the level of the least knowledgeable listener in multiparty conversation [63]. We acknowledge that the small sample size in this study is a limitation, though necessary given the rare incidence of amnesia. To maximize power to detect effects in our amnesia group, we dummy coded the participant group factor such that amnesia was the reference group. In doing so, the main effect of listener is interpreted as the simple effect of listener for patients with amnesia. To detect whether the effect of listener differs for our neurotypical group, we look to the interaction effects. Guidelines for using mixed-effects linear regression models with two binary fixed effects (e.g., listener and participant group, as used here) suggest that four times as many participants are needed to be sufficiently powered to detect an interaction as for a main effect [64]. Thus, although participants in the neurotypical group produce numerically more words, total steps, optional steps, and semantic statements for the child listener, if these listener effects exist, they may not have been large enough to detect with our sample size via the interaction effect, and the lack of effect should be interpreted with caution due to low statistical power. Two neurotypical participants stood out by providing several instances of direct feedback to the child listener (e.g., “Very good!”, “Hey, you spilled it!”). These same participants also gave a name to the child and provided additional context (e.g., “He’s in Cub Scouts,” “It’s Mother’s Day”). Although this behavior was not consistent for all neurotypical participants, it clearly demonstrates the creativity and flexibility involved in imagining another’s perspective and provides support that the picture prompt was effective at eliciting demonstrations intended for a child.

### 4.2. Hippocampal Contributions to Audience Design in Gesture

Gestures are a critical part of narrative demonstrations because they allow the speaker to both show and tell. Despite no overt speech adaptation for the child listener, patients with amnesia produced significantly higher gesture rates for the child compared to the adult listener. This was of the magnitude of about five more gestures per 100 words. This increase in frequency is striking, given that the average speech rate in procedural discourse tasks is 174 words per minute [65]. Although there was no effect of listener for the amnesia group on the likelihood of producing a representative gesture, there was also no significant effect of group, meaning that patients with amnesia, like neurotypical peers, used predominantly iconic gestures in their demonstrations for both adult and child listeners. Thus, not only are patients with amnesia producing more gestures for the child listener, but those gestures are also meaningful and informative, depicting objects and actions related to their speech content. Unexpectedly, we found that patients with amnesia were significantly less likely to produce two-handed gestures than neurotypical participants. Although this could be an indication that patients with amnesia produce less informative gestures than neurotypical peers, future work should investigate whether speakers do indeed modulate gesture handedness for communicative intent and its effect on listener visual attention and comprehension of a message. 

Of note, Patient 1951, whose lesion extends beyond the hippocampus into bilateral medial temporal lobe more broadly, performs within normal limits on standardized tests of language (Boston Naming Test and Token Test) and intelligence (WAIS-III), like the other three patients with amnesia. The patients all demonstrate a specific and selective impairment in memory. Figures displaying patient data include unique markers for each individual with amnesia, and Patient 1951 performs in line and often numerically higher than patients with amnesia who have more focal hippocampal damage (e.g., of patients with amnesia, Patient 1951 produced the second highest gesture rate and proportion of representative gestures, despite his more extensive damage). This suggests that his data, and more extensive lesions, are not driving the presence or lack of any effects across analyses.

Here, we focus on the significant findings in amnesia, where adaptation in gesture rate, but not speech, provides evidence of audience design. Similarly, in collaborative referential barrier games, patients with amnesia mark common ground status in gesture via gesture height [44] but inconsistently in speech, arriving at concise shared labels but not marking common ground consistently with definite references [30]. A growing body of work suggests that common ground is not supported by a single memory system [62] and that gesture use may be supported by the declarative memory system for some tasks [34] while leveraging non-declarative memory in others [40,42,43,66]. Contributions of particular memory systems may depend on the task demands. Increasing evidence suggests that the hippocampus is linked to the flexible use of language and online language processing that allows us to imagine, explore, and navigate complex social interactions [4,5,29], whereas nondeclarative memory may support more implicit or automatic processes. 

### 4.3. Gesture as a Window into Social Cognition and Social Communication

These results suggest that gesture may uniquely reveal evidence of common ground, even in the absence of verbally demonstrated common ground or intact declarative memory. The nonverbal nature of gesture lends itself to affordances that reflect these conceptual representations, with emerging evidence that it may do so by leveraging neural mechanisms outside of the hippocampal declarative memory system. Indeed, gesture may act as a bridge between declarative and nondeclarative knowledge [67], where speech and gesture have a complementary function in supporting comprehension, learning, and memory [68,69,70,71,72]. Producing gestures paired with naming novel words even improves recognition memory in patients with amnesia, who otherwise are profoundly impaired at learning new words [43]. This synergy between speech and gesture has exciting potential implications for leveraging gesture to support learning for those with memory impairments or other cognitive-communication deficits [73]. Indeed, while the current study suggests that patients with amnesia may produce gestures that are intended to benefit the listener, the frequency and meaningfulness of the gestures they produced in this study suggest that those with memory impairments may have access to the speaker-oriented benefits of gesture as well, where representational gestures directly interact with cognition to help speakers activate, manipulate, package, and explore spatio-motoric information to facilitate speaking and thinking [74].

In addition to the evidence that gesture taps into implicit and nonverbal knowledge states, another salient feature of gesture is that it is motoric, and thus, may facilitate and reflect learning via procedural memory mechanisms. Indeed, the gestures of neurotypical speakers reflect their prior experience observing and producing actions [16], whereas the gestures of patients with Parkinson’s disease who have impaired procedural memory do not [40], and research in neurotypical participants suggests that perceiving gesture facilitates learning by activating the listener’s own motor system [75]. The Gesture as Simulated Action framework proposes that gestures manifest from the motor activity that occurs when people think and talk about actions and imagery [35]. According to this framework, for a gesture to be produced, it must activate the motor system with enough strength to exceed the speaker’s gesture threshold. This framework accounts for differences in gesture production by individuals and across contexts and suggests that the form of the gesture produced reflects the underlying mental simulations. Thus, gesture production appears to be supported, not only from declarative memory representations dependent on the hippocampus [34], but also from broader neural mechanisms that reflect our implicit knowledge, sensorimotor experiences, and mental imagery. 

The data here, together with other studies linking memory systems to unique aspects of adaptive and flexible communication, suggest that a larger neural network supports audience design beyond the well-established role of the frontal lobe. It is an area for further investigation how memory supports perspective taking to facilitate aspects of audience design. When audience design involves taking the listener’s perspective, it may share a common neural system with similar abilities such as mentalizing, theory of mind, and prospection [2]. While regions in the prefrontal cortex have a well-established role in perspective taking [76,77,78], there is converging evidence that bidirectional connections between the prefrontal cortex and medial temporal lobe subsystems, including the hippocampus, form a larger network of brain regions that work in concert to support social cognition [2,3,79,80]. In addition to providing an explicit record of past events and conversations that contribute to knowledge about who knows what, the hippocampal declarative memory system contributes to representational flexibility [81]. We propose that this representational flexibility allows speakers to reconstruct prior knowledge to not only recall the past, but also imagine the future [56,82,83,84] and the mental states of others, which are critical ingredients in audience design. Thus, future work should consider the memory demands involved in audience design and social cognition more broadly. 

### 4.4. Limitations and Methodological Considerations for Future Studies

There are limitations to the current study. First, our design compared an imaginary child listener elicited with a picture prompt to a live adult listener. Although there is precedent for using invisible listeners [85,86], imaginary listeners [19,87], and picture prompts to elicit responses [88], our design introduced a systematic difference between experimental conditions. Thus, we were measuring how participants adapt their communication when asked to explicitly imagine a child listener compared to the baseline explanations they provided to the adult experimenter. Using a picture prompt for the child listener was necessary in the current study due to geographical constraints of the amnesia group. Nevertheless, we do acknowledge that asking participants to deliberately imagine that they are speaking to a child listener may differ from the more spontaneous and automatic processes of audience design that occur in real-life complex communication contexts, and thus, limits the generalizability of the current findings. The use of an imaginary child listener may have placed additional demands on the hippocampus. It is possible that patients with amnesia would have shown evidence of audience design in speech if speaking to a live rather than imaginary child listener, an avenue for future investigation. 

Another limitation of the current study is the order of conditions. All participants demonstrated the tasks first to the adult listener and then to the child listener 30 min later. Due to our small sample size, we made this decision in an attempt to reduce variability and noise in the data. In this experiment, order effects would most likely present as practice or repetition effects. When repeating a story, speakers use fewer words [13] and gesture less frequently [20]. Therefore, if there are repetition effects in the current study, we would expect that participants would produce fewer words and gestures in the child listener condition, which always came second. Although neurotypical participants produced numerically more words and instructional steps to the child listener, it is possible that order effects attenuated the effect of listener on speech and gesture adaptation. Order effects are less of a concern for patients with amnesia who, after the 30 min delay between conditions, lack explicit episodic memory for having previously described the tasks. 

We acknowledge that these design decisions may have influenced the present results. Discussions about the merits or challenges and limitations of certain design features provide an opportunity to increase methodological rigor in the study of gesture and is of particular importance as the study of co-speech gesture is rapidly being extended to clinical populations with neurogenic disorders [41,73,89,90,91]. Although the small sample size and lack of significant interactions with the neurotypical group temper our ability to make strong generalizations about the function of the hippocampus in multimodal audience design, this work provides preliminary evidence that adaptation in gesture may be resilient to damage to the hippocampal declarative memory system and is an example of the utility of gesture to act as a window into the social cognitive processes of individuals with neurologic disorders. In this way, these data provide a starting point for the development of subsequent hypothesis formulation around the role of memory in gesture and audience design. Indeed, these findings and studying individuals with hippocampal amnesia more broadly can provide insights into the communicative behavior of other clinical populations for whom hippocampal pathology is common (e.g., Alzheimer’s disease, traumatic brain injury, schizophrenia), where the role of gesture in communication and cognition is largely unexplored.

## 5. Conclusions

In summary, when providing narrative demonstrations for everyday tasks, patients with amnesia showed no adaptation for the child listener relative to an adult listener in speech, but they did show adaptation in gesture, producing gestures at significantly higher rates for the child than the adult listener. Patients with amnesia did not significantly differ from neurotypical participants in their rate of gesture production or the likelihood of producing iconic gestures, suggesting that the production of meaningful and communicative gestures in procedural discourse did not depend on the hippocampal declarative memory system. We propose that multiple memory systems contribute to multimodal audience design, where the nonverbal and motoric properties of gesture may make it better suited to reflect implicit knowledge or nondeclarative memory representations. Studying gesture production and comprehension in clinical patients with neurologic and communication impairments may provide unique insights into the social cognitive processes that underly rich dynamic communication contexts, where hands may reveal not only the richness of our own knowledge and representational systems, but also our shared or inferred knowledge of others.

## Figures and Tables

**Table 1:** Demographic, Neuroanatomical, and Neuropsychological Characteristics of Participants with Hippocampal Amnesia.

								Neuropsychological Scores			
	Demographic Characteristics						Anatomical	Intelligence	Memory	Language	
Participant	Sex	Birth Year	Hand.	Ed.	Etiology	Damage	HC Volume	WAIS-III FSIQ	WMS-III GMI	BNT	TT
1846	F	1963	R	14	Anoxia	Bilateral HC	**−4.23**	84	**57**	43	41
2363	M	1956	R	18	Anoxia	Bilateral HC	**−2.64**	98	**73**	58	44
2563	M	1955	L	16	Anoxia	Bilateral HC	N/A	102	**75**	52	44
1951	M	1952	R	16	HSE	Bilateral HC + MTL	**−8.10**	106	**57**	49	44
Group Mean							**−5.0**	97.5	**65.5**	50.5	43.3

Note: Hand. = handedness. Ed. = years of completed education. HSE = Herpes Simplex Encephalitis. HC = hippocampus. +MTL = damage extending into the greater medial temporal lobes. N/A = no available data. Volumetric data are z-scores as measured through high-resolution volumetric MRI and compared to a matched neurotypical group [45,46]. WAIS-III FSIQ = Wechsler Adult Intelligence Scale–III Full Scale Intelligence Quotient (mean: 100, standard deviation: 15). WMS-III GMI = Wechsler Memory Scale–III General Memory Index (mean: 100, standard deviation: 10). BNT = Boston Naming Test (max score: 60). TT = Token Test (max score: 44). Bolded scores are impaired as defined as two or more standard deviations below normative data.

**Table 2:** Sample Demonstrations of Heating up Leftovers in the Microwave for Adult and Child Listeners by a Patient with Amnesia and a Neurotypical Participant.

Amnesia		Neurotypical	
Adult	Child	Adult	Child
Um, depending on what leftovers dictates the time. Okay, so we’ll just say something simple—mac and cheese. Set it for about forty-five seconds for a dish of mac and cheese. Open the door and put it, uh, put the mac and cheese in a bowl, a glass bowl. It has to be a glass bowl or a Pyrex bowl, one of the two, and, uh, nonmetal. Then you put that into the microwave. Close the door. Time is already set. So, you push start, and then it rings when it’s finished.	First you take a, uh, Pyrex plate, not metal. And you, uh, put your leftovers on the plate that you take out of the refrigerator. And you put it on the amount of time you want to heat it which in this case is probably about a minute and a half or two. Take it out of the microwave. Stir it up to make sure it’s equally heated, and then check to see if it’s the right temperature. If it’s not, then you reheat it, for another, say, fifteen seconds. Once it’s hot enough, then you can eat it.	Um, I would take the leftovers, uh, out of the refrigerator. I would, uh, put them in a microwave-safe vessel. Uh, I would place that vessel… Uh, I would carry that vessel to the microwave. I would open the microwave door. I would place the vessel into the microwave. I would close the door. I would use the keypad to select an appropriate amount of time. And I would push the start button.	So, I want you to go to the refrigerator and take out the leftovers that you want to eat. And those are in a plastic dish, and we can’t put plastic in the microwave. So, I want you to take your leftovers, however much you want to eat, and put them in this glass bowl. Now I want you to take the leftovers that you’ve put in the bowl, and open the microwave door, and put them inside. Close the door. And then I think we need to cook these for one minute. So, push one-zero-zero. And then I want you to push start, and they will be cooking. And when they’re done, you can open the door and take them out.

**Figure A1:**
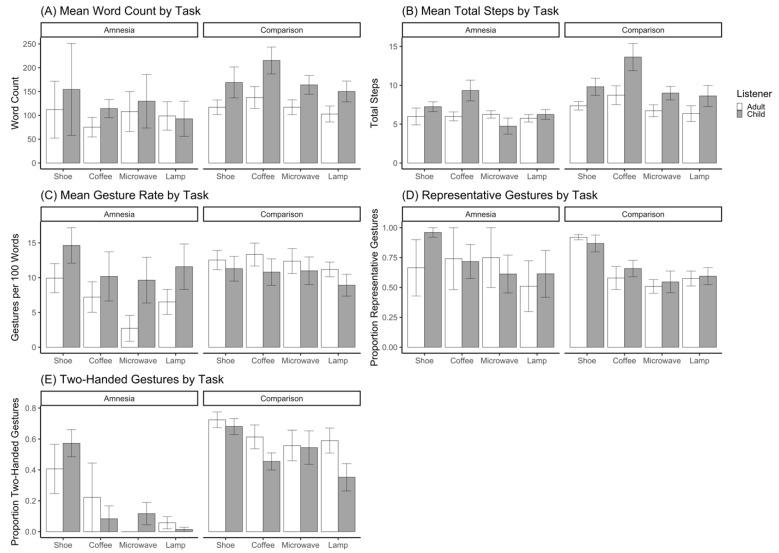
Mean values for speech and gesture outcome variables, stratified by task, group, and listener. Bars represent standard error of the mean.

## Data Availability

The data presented in this study are available on request from the corresponding author.

## Supplementary Materials

The following supporting information can be downloaded at: https://www.mdpi.com/article/10.3390/brainsci12081082/s1, Supplementary Materials A: Gesture Coding Guide; Supplementary Materials B: Speech Coding Guide.

## Appendix A. Mixed Effect Models

**Table A1:** Word count as a function of Group (Amnesia vs. Neurotypical) and Listener (Adult vs. Child); Negative binomial family mixed effect model, fit by maximum likelihood (Laplace Approximation).

Fixed Effects		Estimate	Std. Error	z Value	Pr (>|z|)
(Intercept)		4.591	0.239	19.252	**<0.001**
Group		0.307	0.269	1.139	0.255
Listener		0.176	0.116	1.514	0.130
Group:Listener		0.196	0.133	1.468	0.142
Random effects		Variance	SD		
Participant	(Intercept)	0.199	0.447		
Task	(Intercept)	0.015	0.121		

**Table A2:** Total steps as a function of Group (Amnesia vs. Neurotypical) and Listener (Adult vs. Child); Poisson family mixed effect model, fit by maximum likelihood (Laplace Approximation).

Fixed Effects		Estimate	Std. Error	z Value	Pr (>|z|)
(Intercept)		1.845	0.164	11.248	**<0.001**
Group		0.264	0.171	1.548	0.122
Listener		0.115	0.144	0.799	0.425
Group:Listener		0.227	0.162	1.404	0.160
Random effects		Variance	SD		
Participant	(Intercept)	0.065	0.256		
Task	(Intercept)	0.021	0.145		

**Table A3:** Essential steps as a function of Group (Amnesia vs. Neurotypical) and Listener (Adult vs. Child); Poisson family mixed effect model, fit by maximum likelihood (Laplace Approximation).

Fixed Effects		Estimate	Std. Error	z Value	Pr (>|z|)
(Intercept)		1.370	0.138	9.899	**<0.001**
Group		0.070	0.104	0.671	0.502
Listener		0.050	0.182	0.273	0.785
Group:Listener		0.045	0.209	0.217	0.828
Random effects		Variance	SD		
Task	(Intercept)	0.043	0.208		

**Table A4:** Optional steps as a function of Group (Amnesia vs. Neurotypical) and Listener (Adult vs. Child); Poisson family mixed effect model, fit by maximum likelihood (Laplace Approximation).

Fixed Effects		Estimate	Std. Error	z Value	Pr (>|z|)
(Intercept)		0.788	0.315	2.505	**<0.001**
Group		0.486	0.293	1.660	0.097
Listener		0.230	0.238	0.965	0.335
Group:Listener		0.362	0.260	1.393	0.164
Random effects		Variance	SD		
Participant	(Intercept)	0.198	0.445		
Task	(Intercept)	0.138	0.371		

**Table A5:** Semantic statements as a function of Group (Amnesia vs. Neurotypical) and Listener (Adult vs. Child); Poisson family mixed effect model, fit by maximum likelihood (Laplace Approximation).

Fixed Effects		Estimate	Std. Error	z Value	Pr (>|z|)
(Intercept)		0.352	0.372	0.946	0.344
Group		0.343	0.362	0.948	0.343
Listener		0.174	0.337	0.515	0.606
Group:Listener		0.332	0.368	0.902	0.367
Random effects		Variance	SD		
Participant	(Intercept)	0.300	0.547		
	Listener	0.097	0.312		
Task	(Intercept)	0.156	0.395		

**Table A6:** Gesture Rate as a function of Group (Amnesia vs. Neurotypical) and Listener (Adult vs. Child); Linear mixed effect model, fit by REML.

Fixed Effects		Estimate	Std. Error	df	t Value	Pr (>|t|)
(Intercept)		9.090	2.064	13.968	4.404	**<0.001**
Group		2.335	2.363	13.142	0.988	0.341
Listener		4.844	2.142	13.320	2.262	**0.041**
Group:Listener		−6.701	2.493	13.160	−2.688	**0.018**
Random effects		Variance	SD			
Participant	(Intercept)	14.818	3.849			
	Listener	11.993	3.463			
Task	(Intercept)	0.618	0.786			

**Table A7:** Gesture Rate as a function of Group (Amnesia vs. Neurotypical) and Listener (Adult vs. Child) with neurotypical group set as reference; linear mixed effect model, fit by REML.

Fixed Effects		Estimate	Std. Error	df	t Value	Pr (>|t|)
(Intercept)		11.425	1.279	14.056	8.933	**<0.001**
Group		−2.335	2.363	13.142	−0.988	0.341
Listener		−1.857	1.276	12.722	−1.456	0.170
Group:Listener		6.701	2.493	13.160	2.688	**0.019**
Random effects		Variance	SD			
Participant	(Intercept)	14.818	3.849			
	Listener	11.993	3.463			
Task	(Intercept)	0.618	0.786			

**Table A8:** Representative gestures as a function of Group (Amnesia vs. Neurotypical) and Listener (Adult vs. Child); binomial family mixed effect model, fit by maximum likelihood (Laplace Approximation).

Fixed Effects		Estimate	Std. Error	z Value	Pr (>|z|)
(Intercept)		0.966	0.635	1.522	0.128
Group		−0.086	0.546	−0.158	0.874
Listener		0.437	0.417	1.048	0.295
Group:Listener		−0.302	0.458	−0.660	0.509
Random effects		Variance	SD		
Participant	(Intercept)	0.716	0.846		
	Listener	0.192	0.438		
Task	(Intercept)	0.702	0.838		

**Table A9:** Two-handed gestures as a function of Group (Amnesia vs. Neurotypical) and Listener (Adult vs. Child); binomial family mixed effect model, fit by maximum likelihood (Laplace Approximation).

Fixed Effects		Estimate	Std. Error	z Value	Pr (>|z|)
(Intercept)		−1.313	0.462	−2.844	**0.004**
Group		1.623	0.436	3.725	**<0.001**
Listener		0.417	0.286	1.456	0.145
Group:Listener		−0.908	0.313	−2.901	**0.004**
Random effects		Variance	SD		
Participant	(Intercept)	0.457	0.676		
Task	(Intercept)	0.278	0.527		

## Appendix B. Differences in Outcome Variables by Task

Table A10 contains mean values for speech and gesture variables, stratified by task and group, to facilitate comparisons of between-task differences. Of note, all participants completed the procedural narrative tasks in the following order: shoe, coffee, microwave, and lamp

**Table A10:** Mean and standard deviation values for outcome variables stratified by task and group.

	Amnesia				Neurotypical			
	Shoe	Coffee	Microwave	Lamp	Shoe	Coffee	Microwave	Lamp
Word Count	133 (150)	95 (37)	119 (93)	96 (62)	143 (87)	176 (93)	141 (63)	127 (67)
Total Steps	6.6 (1.8)	7.7 (2.4)	5.5 (1.7)	6.0 (1.1)	8.6 (3.1)	11.2 (5.5)	7.9 (2.9)	7.5 (4.1)
Gesture Rate	12.3 (5.0)	8.7 (4.8)	6.2 (6.2)	9.0 (5.6)	11.9 (5.2)	12.1 (5.9)	11.7 (6.2)	10.1 (4.5)
Representative Gestures	0.81 (0.35)	0.73 (0.32)	0.66 (0.30)	0.56 (0.38)	0.89 (0.16)	0.62 (0.27)	0.53 (0.25)	0.58 (0.21)
Two-Handed Gestures	0.49 (0.26)	0.15 (0.27)	0.08 (0.13)	0.04 (0.06)	0.70 (0.16)	0.54 (0.23)	0.55 (0.33)	0.48 (0.29)

Word Count = Total number of words produced; Total Steps = Total number of steps produced; Gesture Rate = Gestures per 100 words; Representative Gestures = Proportion of total gestures that were representative (iconic + deictic); Two-Handed Gestures = Proportion of total gestures produced with both hands. Values in parentheses are standard deviations.
