# Supplementary material for: Evidence of Audience Design in Amnesia: Adaptation in Gesture but Not Speech
Source: Brain Sci. 2022 Aug 16;12(8):1082. doi: 10.3390/brainsci12081082 (PMC9405987; doi:10.3390/brainsci12081082)

Supplementary Materials A: Gesture Coding Guide

Setting Up ELAN Environment

- Create new file by adding media file
- Create tiers for "RightHand" and "LeftHand"
- Delete "default" tier

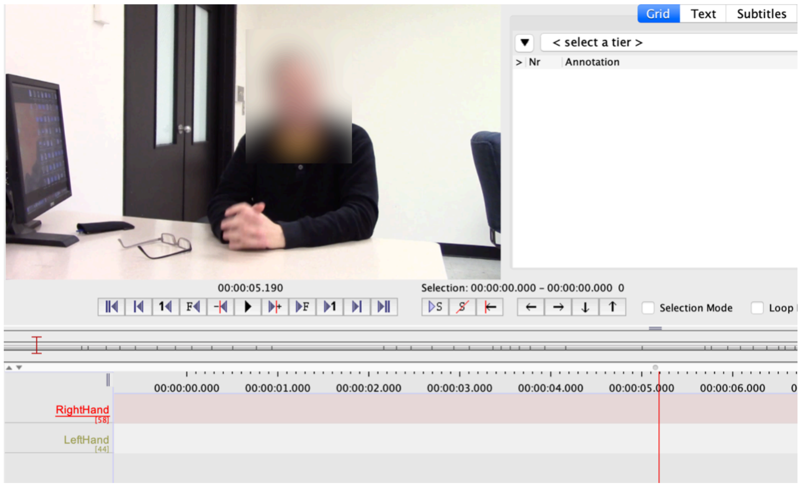

Identifying Gestures

- Co-speech gestures are all hand movements that are semantically or rhythmically linked to accompanying speech. Code only hand movements occurring with speech (i.e., do not code gestures produced when thinking only).
- Gestures *are not* self-adjusting movements such as scratching, fidgeting, adjusting hair/glasses, etc.

Coding Gesture Type (McNeill, 1992)

- Iconic gestures (icon): Gestures that visually depict the shape, size, position, or movement of an object and are semantically related to speech.
- Beat gestures (beat): Gestures that occur in rhythm with speech but are not communicative. These are often very short or repetitive gestures (e.g., baton-like movements, finger or hand taps, wrist flicks or flourishes) and can be subtle. Abstract or pragmatic gestures (e.g., turning hand over in a shrugging gesture) were coded as beat gestures for this study.
- Deictic gestures (deic): Gestures that refer to the location of an object in space, often a pointing gesture.

Annotating Gestures

- Gesture onset: Start coding as soon as hands begin to move.
    - If the speaker raises hands from lap or table and initiates gesture in a smooth continuous movement, start coding from lap.
    - If a participant raises hands and pauses before producing gesture, do not include initial hand movement.
- Gesture offset: Stop coding when the hands pause after completing a gesture.

- If the speaker drops hands after gesture in a continuous movement, include dropping.
- If the speaker completes gesture and pauses before dropping hands, stop coding after completion of gesture form.
- If the speaker produces consecutive gestures, separate each one where the hands pause. Separate gestures that are distinct in form or semantic relation to speech.

- Handedness: Create an annotation for each hand that produced a gesture (left hand, right hand, or both).
  - If speaker only moves one hand, but the non-moving hand is involved in the interpretation of the gesture (e.g., “you cross the right lace [crossing gesture] over the left lace” [keeping the left hand still]), code as two-handed gesture.
  - If speaker has both hands raised but only moves one hand and the non-moving hand is not needed to interpret the gesture, code as a single-handed gesture.
  - Two-handed gestures should overlap entirely in time (i.e., code gestures as a single unit even if one hand starts moving before the other).
  - Two-handed gestures must always be labeled as the same gesture type (i.e., do not code a beat gesture for the right hand and an iconic gesture for the left hand for the same annotation).

Creating annotation

- Click and drag to select section you want to annotate
- Double click on right-hand or left-hand tier
- Name it using four-letter code for gesture type

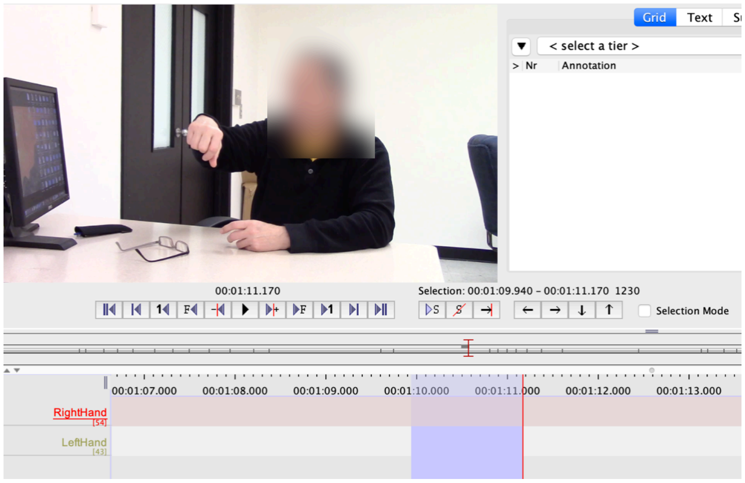

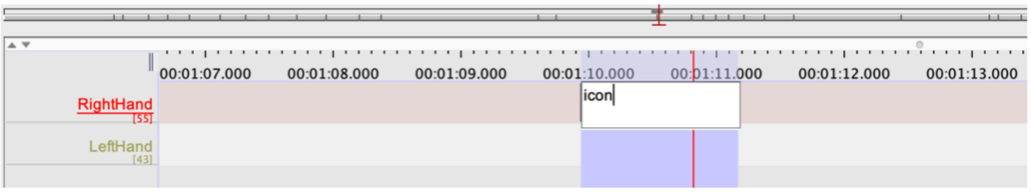

Exporting annotation

- Export tiers as tab-delimited text and select both right- and left-hand tiers
- Output options: select “separate columns for each tier” and “repeat values of annotations spanning other annotations”
- Include time column for: Begin Time and End Time
- Include time format: ss.msec

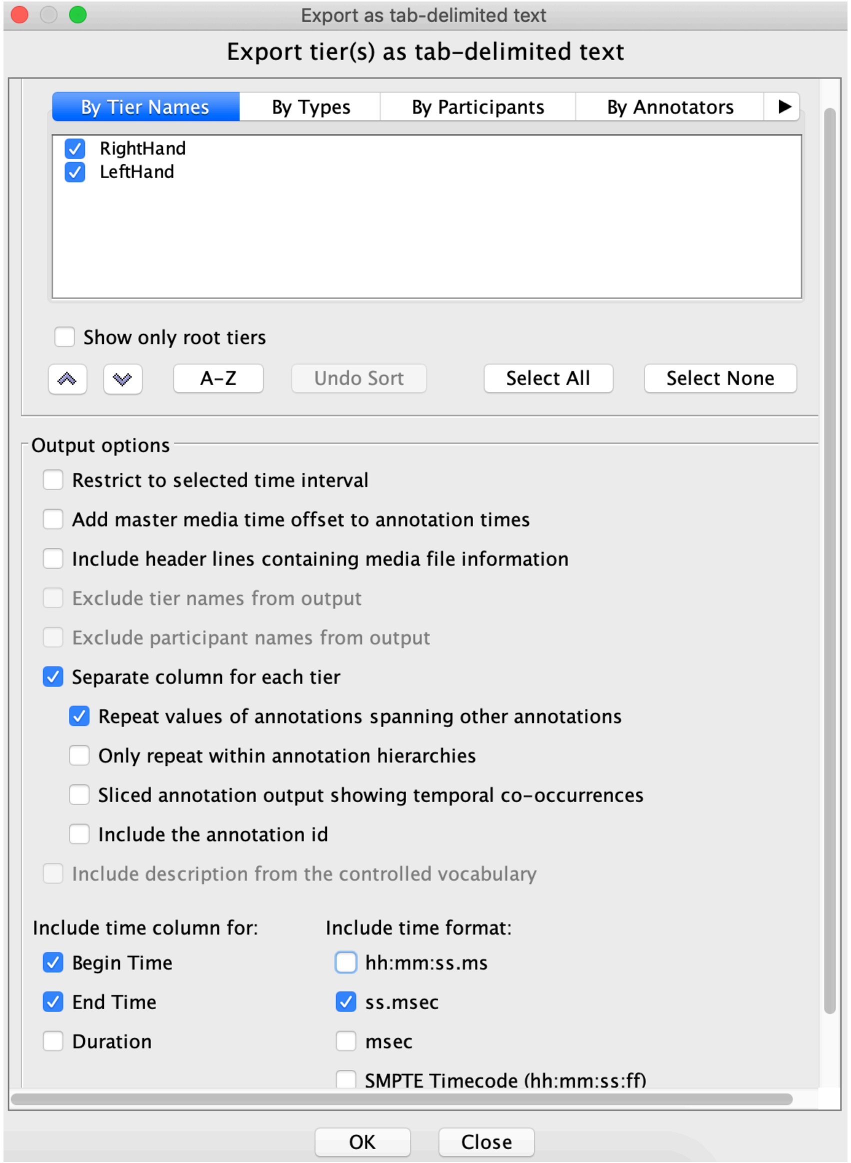

Supplement: Supplementary file 1 [file brainsci-12-01082-s001.zip › Supplementary Materials A.pdf]
