# Supplementary material for: Evidence of Audience Design in Amnesia: Adaptation in Gesture but Not Speech
Source: Brain Sci. 2022 Aug 16;12(8):1082. doi: 10.3390/brainsci12081082 (PMC9405987; doi:10.3390/brainsci12081082)
Supplement: Supplementary file 1 [file brainsci-12-01082-s001.zip › Supplementary Materials B.pdf]

Supplementary Materials B: Speech Coding Guide

Essential Steps: Steps determined *a priori* by consensus of 2 authors that reflect the minimum steps required to successfully complete task

Optional Steps: Instructions to do things that are beyond essential steps.

Semantic Information
Safety: Additional information pertaining to safety
Rationale clauses "So that you don't get burnt when you put the lightbulb in"
Warnings: "This is dangerous." "Don't drop it." "Don't screw it in too tight."

World knowledge/elaboration: providing additional information about the objects or tasks involved.
About objects: "Most coffee machines have a timer." "It'll be red."
About actions: "It screws in counterclockwise."
About personal routines: "In my microwave, I use a splatter shield."
Generalizations: "Most bulbs are 60 watts."
What not to do's: "Leave the shade on." "Don't unplug it."

Locations: "It sits on the burner." "Those are kept in the hallway closet."

Management
Feedback: Direct input to listener
"Do you want it [coffee] to be light or dark?" "That's the wrong foot." "Perfect!" "You spilled a little but we'll clean it up." "You know what a bunny's ear looks like." "Did I tell you about the water?"

Task management: Organizing task, stating assumptions, or metacognitive statements
"We're going to change the lightbulb in the table lamp." "It's already plugged in." "and then you should probably have a good bow." "Let's say we're making mac and cheese." "That's it, I think." "It's so much harder when you're not actually doing it."

Repetition
Repetition: a step that has been repeated (note: doesn't count as repetition if the same step represents a different stage in the task – e.g., tightening shoe multiple times throughout tying process)

Restarts: incomplete thoughts, abandoned utterances

*See below for common examples from participant transcripts for each category

Essential Steps

**Coffee**	**Lamp**
1. Get the coffee<br>2. Put a filter in<br>3. Put the coffee in<br>4. Put water in<br>5. Put a container underneath<br>6. Turn on the machine	1. Unplug lamp/make sure it's off<br>2. Unscrew the lightbulb<br>3. Get/find a new lightbulb<br>4. Screw new bulb in<br>5. Turn on light/Check to make sure it works
**Microwave**	**Shoe**
1. Get food from fridge<br>2. Put it in microwave<br>3. Set time<br>4. Hit start	1. Pull laces tight<br>2. Cross laces<br>3. Fold one under<br>4. Tighten<br>5. Create a loop<br>6. Wrap a string around the loop<br>7. Poke through space<br>8. Pull to tighten

Optional Steps

**Coffee**	**Lamp**
Go into the kitchen	Go over to the lamp
Grind the beans	Get an adult
Get the coffee pot out	Check whether bulb is burnt out
Rinse out coffee pot	Unscrew shade/remove screw
Get chair to stand on	Remove shade
Open tray/lid	Set shade down
Remove the basket	Dump out bugs
Throw old filter away	Get a pair of gloves
Get a coffee filter	Check wattage on bulb
Open coffee filter up	Set old bulb down
Close tray/lid	Throw old bulb away
Read instructions	Buy a new bulb
Open the lid to the coffee	Go to the cupboard/closet
Scoop/measure the coffee	Put bulb into socket
Take the pot to the sink	Put shade back on lamp
Turn on the water	Screw shade back in
Fill coffee pot with water	Plug the lamp back in
Take it back to the coffee maker	Turn lamp off again
Plug in the coffee pot	Put supplies away
Get sugar/cream or other supplies	
Drink it	

**Microwave**	**Shoe**
Go to the fridge	Sit down
Get out a plate/bowl	Loosen the laces
Put food on plate or in container	Put the shoe on
Read the instructions	Pull out the tongue
Loosen lid	Pick up the laces
Carry container to microwave	Make laces same length
Open the microwave door	Hold lace between fingers
Put a shield/paper towel on top	Hold knot down with your finger
Shut the microwave door	Switch hands
Set the temperature/power	Grab the lace with […] hand
Wait for it to heat up	Make a loop with the other string
Check temperature	Pull finger out of way
Stir food	Adjust size of bow
Reheat	
Take it out	
Set it on table	
Eat it	

Semantic Information

**Coffee**	**Lamp**
The coffee pot is in the kitchen	The lightbulb might be hot
It might be unplugged	I always unplug it
Don’t touch the metal prongs	Don’t electrocute yourself
There’s a tray that pulls out	You light should be turned off
Inside there’s a basket	I leave the light shade on
The basket holds the filter	There might be bugs
It will probably have an old filter in it	You can use gloves/pliers
That’s where the coffee goes	It screws out by rotating
The coffee is kept […]	You turn it […] direction to (un)screw it
It’s already ground up	The lightbulbs are glass
There’s a scoop inside	You have to be careful
Use one tbsp per cup	Don’t break the bulb/drop it
More coffee makes it stronger	Lightbulbs come in different wattages/kinds
It is a glass pot	It’s usually […] watts
There are measurement readings on the side	There is a number on the top
There’s a lid that goes on top	You have to get the right one
It’s on the back	They are in the cupboard/drawer
There’s a button that turns it on	It will tighten up
It’s red	Don’t force it
Most coffee machines have a timer	Be careful
The coffee will drip down	Don’t look directly at it
The coffee is ready when the timer goes off	
It takes about […] minutes	
**Microwave**	**Shoe**
Not every container can go in the microwave	You don’t have to put the shoe on
Don’t put metal in the microwave	It should be comfortable on your foot
I usually use a paper plate	There are two laces
I use a spatter shield	The laces are about a foot long
There’s a button that pops the door open	It’s a two-part knot
There’s a rotating table	The first part is an overhand knot/slip knot
The food might come with instructions	It will look like an […]
For (insert food), heat […] seconds	It should be a circle
Make sure you don’t do minutes	They look like bunny ears
There is a number pad	There will be an opening in the loop
There’s a button that says […]	It’s like tying a package
The microwave will ding when it’s done	The loops might be uneven
Cook longer for big items	You can untie it by pulling
It might make a mess	
There’s a viewing window	
When it’s hot, you eat it	
There might be steam	
Don’t burn your mouth	

Management

**Coffee**	**Lamp**
We're going to make coffee	I would tell an 8-yo…
The first step is…	We're going to change a lightbulb
Can I do an automatic?	The lightbulb burnt out last night
I use a […] coffee maker	I'm assuming I have the right lightbulb
I'm assuming the coffee pot is clean	I'm assuming the lamp has a shade
It's already plugged in	I'm assuming the bulb is no longer working
Did I tell you about the water?	I don't know […]
Let's pretend…	We're going to make it easy
I think you need water	Do you see that […]?
I don't know about […]	You have the bulb in your hand
I forgot a step	What size is it?
I'll move the chair	I left a step out
Very good	I've got a lightbulb here
You spilled a little	Good job
There you go	There you go
You don't have to worry about that	And then you should be done
And then it will come through	
And then you've made coffee	
**Microwave**	**Shoe**
We're going to make lunch	We're going to tie a shoe
I'm assuming it's in a microwave container	I'm assuming the shoe is laced
Let's say you're making […] food	So you've got your shoe on your foot
I'm going to show you what buttons to push	That's the wrong foot
The time is already set	Let's start with […] foot
Does it seem hot?	He's right-handed
We might need a little more time	I do it the easy way
It's not so easy	We're going to do the bunny ears method
Hopefully no smoke will come out	You know what a bunny's ear looks like
And then the food is heated/you're done	He's in cub scouts
And there you go	Now we're going to […]
Let's eat	Now for the main part
	So you have your loop
	Very good
	I skipped a step
	This is much harder when you're not doing it
	Then you'll have a bow/shoe is tied/ That's it.
